# Neural Correlates of Voice Learning with Distinctive and Non-Distinctive Faces

**DOI:** 10.3390/brainsci13040637

**Published:** 2023-04-07

**Authors:** Romi Zäske, Jürgen M. Kaufmann, Stefan R. Schweinberger

**Affiliations:** 1 Department of Experimental Otorhinolaryngology, Jena University Hospital, Stoystraße 3, 07743 Jena, Germany; 2 Department for General Psychology and Cognitive Neuroscience, Institute of Psychology, Friedrich Schiller University of Jena, Am Steiger 3/1, 07743 Jena, Germany; 3 Voice Research Unit, Friedrich Schiller University of Jena, Leutragraben 1, 07743 Jena, Germany

**Keywords:** voice recognition, audiovisual integration, distinctiveness, N250

## Abstract

Recognizing people from their voices may be facilitated by a voice’s distinctiveness, in a manner similar to that which has been reported for faces. However, little is known about the neural time-course of voice learning and the role of facial information in voice learning. Based on evidence for audiovisual integration in the recognition of familiar people, we studied the behavioral and electrophysiological correlates of voice learning associated with distinctive or non-distinctive faces. We repeated twelve unfamiliar voices uttering short sentences, together with either distinctive or non-distinctive faces (depicted before and during voice presentation) in six learning-test cycles. During learning, distinctive faces increased early visually-evoked (N170, P200, N250) potentials relative to non-distinctive faces, and face distinctiveness modulated voice-elicited slow EEG activity at the occipito–temporal and fronto-central electrodes. At the test, unimodally-presented voices previously learned with distinctive faces were classified more quickly than were voices learned with non-distinctive faces, and also more quickly than novel voices. Moreover, voices previously learned with faces elicited an N250-like component that was similar in topography to that typically observed for facial stimuli. The preliminary source localization of this voice-induced N250 was compatible with a source in the fusiform gyrus. Taken together, our findings provide support for a theory of early interaction between voice and face processing areas during both learning and voice recognition.

## 1. Introduction

Current models of a person perception specify how visual and auditory information from faces and voices are combined [1]. However, the exact time-course of audio-visual face-voice-integration remains a matter of debate. For instance, using functional magnetic resonance imaging (fMRI), von Kriegstein et al. [2] showed that unimodal presentation of familiar voices causes activation in areas implicated in face-processing. However, due to the limited temporal resolution of fMRI, it remains unclear whether this activation is mediated by an early (perceptual) locus of face-voice integration, in addition to later top-down processes. In the present study we used the excellent temporal resolution of event-related potentials (ERPs) to investigate this issue. Based on findings that face learning is improved for distinctive as compared to non-distinctive faces (e.g., [3]), we tested whether the recognition of *voices* may also be facilitated by audio-visual learning with distinctive vs. non-distinctive faces. We considered that early differences (<300 ms) for unimodal test voices that have previously been learned with distinctive or non-distinctive faces would support the notion of an early integration of audio-visual person identity information.

Once we are familiar with people, it is usually easy to recognize them by means of their voice alone (e.g., [4]). At the same time, we may be able to put a face to a known voice, provided that both types of information are stored in long-term memory. When getting to know new people, we can typically exploit information from at least two modalities at once, vision and audition [2]. By means of audio-visual integration (AVI), our brains can extract and combine vocal and facial cues. AVI occurs during learning of unfamiliar people and recognition of familiar people and is achieved via multimodal identity representations in long-term memory. This is evidenced by brain-imaging data showing functional and structural connections between a voice and face-sensitive areas in the brain (reviewed in [5]). Specifically, it has been demonstrated that voices alone [2] can activate the fusiform face area (FFA) for personally familiar speakers, as well as for learned, beforehand-unfamiliar, speakers [6]. Moreover, the existence of fiber tracts connecting the FFA with voice-sensitive areas in the superior temporal sulcus (STS) has also been demonstrated [7]. Despite this indirect evidence for an early (perceptual) cross-talk between voice and face areas during voice recognition, it remains possible that face-voice integration during voice learning and recognition is only mediated by a later (post-perceptual) stage, as implied by some classical models [8]. Evidence from electrophysiological measures with high temporal resolution, such as the electroencephalogram (EEG) and event-related potentials (ERPs), is clearly helpful in distinguishing between these possibilities.

While personal recognition from voices is less reliable than from faces [1,9,10], a few exposures to brief-sentence stimuli can suffice to recognize a previously unfamiliar voice in old/new tasks [11,12,13,14]. Although we can learn new voices in the absence of faces, a more common scenario is that we are exposed to both domains simultaneously. Therefore, studying the role of facial information for voice memory has longstanding traditional grounds. Essentially, while most studies show an effect of the presence of faces on subsequent voice recognition of unfamiliar speakers, it is controversial whether faces facilitate [15] or hamper [16,17,18,19,20] voice learning, compared to unimodal voice learning. (For possible factors mediating the effect, see [13,16,21].) While face-associated benefits for voice memory might be facilitated by redundant dynamic information from voices and seen lip-movements [13,22,23,24], face-induced costs have been explained in terms of ‘face-overshadowing’, i.e., distraction from voices during learning. The neural mechanisms underlying audio-visual voice learning, however, are still poorly understood, as both modalities are usually addressed separately in EEG research. (For an overview, see [25].) For instance, in a typical learning study, participants are asked to memorize sets of either voices [11] or faces [3] for a subsequent unimodal recognition test. Learning conditions are manipulated between sets, depending on the research question. At the test, participants are presented with learned and novel stimuli which they then categorize as old or new. Successful learning is usually defined by an above-chance recognition of learned identities which generalizes to previously unseen pictures or unheard utterances of those learned identities.

Moreover, while the presence of faces per se plays a role for audio-visual voice learning, there is hardly any data on whether and how certain facial characteristics affect voice memory. Behavioral research showed that the ‘face-overshadowing effect’ in voice recognition following voice–face learning is eliminated when the face is inverted during learning [16]. While this provides evidence that the negative impact of faces on voice processing can be nullified by disrupting facial identity recognition, the question remains whether certain facial characteristics can *facilitate* voice processing. For instance, would manipulations that promote face recognition also promote audio-visual voice learning? Specifically, we here ask whether it is easier to remember a voice which has been paired with a distinctive, rather than a non-distinctive, face.

Distinctiveness is a potent characteristic that has been shown to facilitate face learning in recognition memory paradigms [3,26], voice recognition [4,27] and unfamiliar voice matching [28], respectively. Distinctiveness of a face or voice can be assessed by asking participants to what extent a stimulus would stand out in a crowd, or to what extent a stimulus deviates from other known stimuli [29].

When asking how face memory can be affected by concomitant voice information, there is little evidence overall for a facilitative effect of voices on face encoding or memory [30]. Vocal distinctiveness, however, may help when matching unfamiliar faces to voices [31], suggesting that voice-distinctiveness may facilitate AVI under certain conditions. Moreover, Bülthoff et al. [32] studied long-term memory for unfamiliar faces which had been paired with either distinctive or non-distinctive voices during study. At the test, recognition accuracy for faces was higher when they had been previously paired with distinctive, as compared to non-distinctive, voices. In a control condition with sounds instead of voices, sound distinctiveness had no effect on subsequent face recognition. With respect to the issue of *when* a speaker’s identity is integrated across modalities, the same group discussed possible evidence for an early perceptual locus of the voice-distinctiveness effect on face memory [33]. In a face-familiarity task following unfamiliar face primes paired with unfamiliar voices, they observed cross-modal behavioral-priming effects. Consistent with their previous study on face learning, priming effects were most pronounced when distinctive, rather than non-distinctive, voices had accompanied the face primes. Together, these findings could hint at the possibility that identity information is integrated relatively early in a multisensory representation in long-term memory. Moreover, distinctiveness may be a sufficiently potent perceptual quality to elicit cross-modal effects in person-memory.

Electrophysiological research into unimodal face perception has revealed systematic correlates of facial distinctiveness in ERPs. The most consistent finding is that distinctive faces elicit a smaller occipito–temporal P200 response than do typical faces [3,34], (for review see [35]), and therefore the distinctive faces affect an ERP response that is sensitive to the deviation of a face from an observer’s perceptual norm [36]. In addition, distinctive faces can elicit larger occipito–temporal negativities in the preceding N170 (presumably reflecting face detection and structural encoding; [37,38]) as well as the subsequent N250 (presumably reflecting face identity processing; [39]). While these components are potentially relevant for the question of early face-voice integration, effects in the N250 time-window may be particularly relevant for cross-modal benefits to speaker identification. Distinctive faces also elicit a larger centro-parietal late positive complex (LPC; [26,40]), which presumably reflects post-perceptual, semantic processing of person-identity. As a further visual component we consider the occipital P100 which reflects early sensory processing and is modulated by selective attention [41]. It is currently unclear whether manipulations of face-distinctiveness via face-caricaturing affect the P100 [42,43].

In terms of auditorily-evoked potentials elicited by voices, we will analyze the obligatory fronto-central N1 and P2 responses. Both reflect an early sensory processing of sound and are sensitive to attentional processes (e.g., [44]) and sound repetitions, including voice repetitions [45,46,47,48,49]. Interestingly, one study which investigated cross-modal (face-to-voice) identity priming found that information conveyed by the face can modulate sensory processing of a subsequent voice in terms of the N1 amplitude [50]. Thus, N1 and P2 are potentially sensitive to voice-learning effects and even very early cross-modal effects. Finally, we will analyze later (>650 ms) occipito–temporal sustained activity (SA) in response to unimodal as well as to audiovisual stimuli. Note that for reasons of readability, we use SA as an umbrella term for both negative- and positive-going deflections. With respect to unimodal face processing, modulations of occipito–temporal negativity have been previously described in the context of face encoding into memory [51], familiar face recognition [52], and also as a correlate of familiar face distinctiveness [36]. In the context of audition, SA has been described as a long-lasting baseline shift in response to sustained auditory stimuli [53,54]. With respect to voices, the SA appeared as a negativity over frontal and central sites and as a positivity over parietal electrodes and has been reported to be sensitive to voice familiarity [53].

In the present study, we considered (1) whether voice memory can be affected by facial distinctiveness in a situation in which either distinctive or non-distinctive faces are paired with voices during learning, and (2) whether brain responses (as quantified with ERP measures) in subsequent unimodal voice recognition tests are modulated depending on whether the same voices had been learned with distinctive or non-distinctive faces.

## 2. Materials and Methods

### 2.1. Participants

Our final sample consisted of 28 student participants (23 female, 5 male, all right-handed and native speakers of German, *M*_age_ = 22.6 years, *SD* = 3.1, and age range = 19–31 years). None reported any hearing difficulties or prior familiarity with any of the voices used in the experiment. From the initial 32 participants, we excluded n = 4 due to poor EEG data quality (n = 3) and technical issues during EEG recording (n = 1). Participants received course credit and gave written informed consent. The study was conducted in accordance with the Declaration of Helsinki and was approved by the Ethics Committee of the Friedrich Schiller University of Jena. Note that a sample size between 20 and 30 tends to be common for this type of study (e.g., [3,11]), and that the present sample size tested was determined by the availability of resources and the practical restraints of the study. Due to the novelty of the present design and the absence of known effect sizes from previously published research, performing an *a priori* power analysis would not have been possible without further assumptions. In order to identify a medium-sized effect (f = 0.25) in a within-subjects design for an experimental variable with three levels with a power of 0.80 and an alpha-level of 0.05, an exploratory analysis with GPower 3.1 [55] suggests a minimum of *N* = 28. On that basis, we think that the present study was adequately powered to identify medium-sized or large (but not necessarily small) effects of interest.

### 2.2. Stimuli

#### 2.2.1. Voices

We recorded voices of 24 unfamiliar and native speakers of German (12 female and 12 male), aged 18–22 years, who uttered three German sentences. The stimuli had been recorded in the context of a more extended protocol, in a sound-attenuated room, in a method analogous to a previous study [22]. High-quality voice clips were obtained with a Sennheiser™ MD-421 dynamic microphone placed in front of the speaker, and digitized at 44.1 kHz and 16-bit resolution (mono). Speakers were asked to articulate clearly and evenly. They were initially played a sample recording of a model speaker to encourage similar speech rates across speakers, in order to meet the stimulus requirements of another study. Five recordings per sentence were taken to allow for a choice at the editing stage. For each speaker, one audio sample was then edited using Adobe Audition^TM^ so that the experiment would contain one sentence starting exactly at plosive onset. Voice stimuli were normalized for mean amplitude and presented via headphones at ~60 dB(A), as determined with a Brüel and Kjær Precision Sound Level Meter Type 2206. Two sentences served as stimuli in the learning phases (“Keine Antwort ist auch eine Antwort” [No answer is also an answer] and “Dichter und Denker dachten dasselbe” [Poets and thinkers thought the same]). Across speakers, the sentences had a mean duration of *M* = 2686 ms (*SD* = 305 ms, range = 2059–3109 ms) and *M* = 2891 ms (*SD* = 277 ms, range = 2378–3367 ms), respectively. A third sentence was used in the testing phase only (“Du bist doch, was du denkst” [You are indeed what you think]), with a mean duration of *M* = 2130 ms (*SD* = 192 ms, range = 1747–2463 ms).

To control for perceived vocal distinctiveness, we analyzed distinctiveness ratings of the 24 voices, as obtained in a previous unpublished learning study. Ratings were based on a certain sentence (“Dichter und Denker dachten dasselbe” [Poets and thinkers thought the same]), and were obtained from 24 women (*M*_age_ = 20.8, range 18–39 years) on a 6-point scale (1 for “very ordinary” to 6 for “very distinctive”). Task instructions specified that a voice is distinctive if it stands out among many voices, thus asking for “in-the-crowd-based” distinctiveness, rather than deviation-based distinctiveness (for a distinction of these types of distinctiveness, see [29]). Due to the design of the learning study, each voice was rated by six of the 24 raters. Vocal distinctiveness ranged from 2.17 to 5.17, with a mean distinctiveness of *M* = 3.50. For the present study, we randomly grouped the voices in four gender-balanced sets of six voices each, in order to combine them with the distinctive and non-distinctive face sets in a pseudo-randomized fashion. To make sure that distinctiveness effects during audio-visual learning would be elicited by facial, rather than vocal, distinctiveness, we combined the distinctive and non-distinctive faces within a given experimental version with two voice sets of the same overall distinctiveness. Equal perceived distinctiveness was confirmed by non-significant t-tests (*t*(10) = −0.497, *p* = 0.630 and *t*(10) = −0.076, *p* = 0.941, respectively) for comparisons of voice set#1 vs. voice set#2 (*M* = 3.30 and 3.50, respectively) and of voice set#3 vs. voice set#4 (*M* = 3.62 and 3.58, respectively).

#### 2.2.2. Faces

As faces, we selected one photo of each of 12 unfamiliar individuals (6 female and 6 male) from the Glasgow Unfamiliar Face Database (GUFD; [56]) and the Facial Recognition Technology (FERET) database [57,58], half of which had been rated as distinctive and non-distinctive in a previous study [26]. Ratings were based on 10 young adult raters (7 females, 3 males, *M*_age_ = 23.3, range 20–28 years) rating 2 photo versions of 335 identities (670 photos) on a 6-point distinctiveness scale (1 = typical, 6 = distinctive), asking for “face-in-the-crowd” distinctiveness. We selected six facial identities (3 female and 3 male) with the highest and lowest mean distinctiveness ratings, respectively. Means were calculated across raters and the two photos for each identity. We considered only faces of Caucasian appearance which received similar ratings across their two photo versions (i.e., less than 1 rating point apart on average). For distinctive face identities, we selected the photo version which received the higher rating of the two photos, while for non-distinctive face identities, we selected the version with the lower rating. This was to maximize the potential effect of distinctiveness on face-voice learning. Mean distinctiveness of the selected faces was *M* = 1.79 and *M* = 4.64 for the non-distinctive and distinctive face sets, respectively.

### 2.3. Procedure

Participants were tested individually in a dimly lit, sound-attenuated booth. Instructions were presented in writing on a computer screen to minimize interference from the experimenter’s voice. Participants underwent six learning-test cycles, each comprising a learning phase with 12 audio-visual trials and a subsequent test phase with 24 voice-only trials. Across the six cycles we repeated the same learning and test stimuli in a randomized order. Learning phases consisted of six trials per learning condition (distinctive and non-distinctive faces), with one speaker per trial. Thus, there were 36 trials per learning condition overall (6 learning phases × 6 learning trials per condition). Faces and voices were gender-matched on learning trials. Test phases consisted of 12 learned target voices and 12 novel voices. Of the 12 target voices, six had been learned with a distinctive face and six with a non-distinctive face. Overall, there were 36 test trials per distinctive and non-distinctive learning condition, respectively (6 test phases × 6 learned target voices per condition), and 72 test trials for the novel voice condition (6 test phases × 12 novel voices).

**Figure 1:**
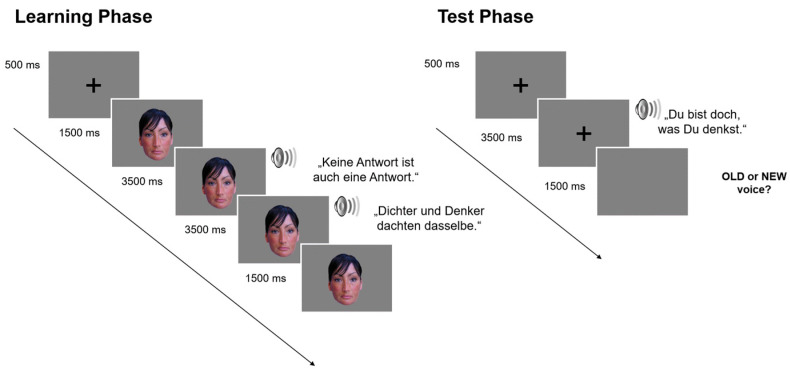
Trial procedure in learning and test phases. In this example, study voices were coupled with distinctive faces.

During learning phases all speakers uttered the same two consecutive sentences once (Figure 1), and a third sentence at the test. This was performed in order to capture voice recognition largely independent of speech content, as an important marker for successful voice learning [11]. Note, however, that there was a degree of phonological overlap between our second learning sentence and our test sentence. Participants were instructed to remember the speakers for a subsequent test. Learning trials started with a black fixation cross on a grey background (500 ms) announcing a face which replaced the cross. One thousand five hundred ms after face onset, two consecutive voice samples (sentence #1 and #2) of the same speaker joined the face, with an SOA of 3500 ms. After 3500 ms from the onset of the second voice sample, the face remained on-screen for another 1500 ms. The total trial duration was thus 10,500 ms, independent of the actual duration of individual voice samples. No responses were required from the participants in the learning trials.

In an immediately ensuing test phase, trials started with a fixation cross (500 ms) which was joined by a test voice uttering one sentence. With an SOA of 3500 ms from voice onset, or after a response was logged, voice presentation terminated, and a grey screen appeared for 1500 ms. Responses were measured from voice onset up to a maximum time of 3500 ms, and participants were asked to classify each test voice as old or new. They were asked to respond as fast and accurately as possible, using their index fingers and the “x” and “m” keys on a computer keyboard (German layout). The maximum total trial duration was thus 5500 ms.

The assignment of keys to old/new responses, the assignment of voices to faces in the learning phases and the designation of voices as targets and foils at the test were counterbalanced in four versions of the experiment. In each experimental version, two voice sets (à six identities) were combined with the two face sets (six distinctive and six non-distinctive voices), while the remaining two voice sets served as foils at the test. To hold vocal distinctiveness constant across audio-visual learning conditions, two voice sets of equal perceived distinctiveness served as learning stimuli in a given experimental version (cf. also Section 2.2, Stimuli).

As there were no additional stimuli available for practice trials, the first test phase was preceded by eight practice trials (four learned and four novel voices), and the other test phases were preceded by four practice trials (two learned and two novel), with stimuli randomly drawn from the 24 available test stimuli. Practice stimuli were excluded from all analyses. The experiment lasted approximately 35 min, with self-paced breaks during instruction screens shown between learning and test phases.

### 2.4. EEG Recordings

A 32-channel EEG was recorded during learning and test phases in an electrically and acoustically shielded room. Data were recorded with sintered Ag/AgCl electrodes mounted on an electrode cap (EasyCapTM, Herrsching-Breitbrunn, Germany) using SynAmps amplifiers (NeuroScan Labs, Sterling, VA, USA), arranged according to the extended 10/20 system at the scalp positions of Fz, Cz, Pz, Iz, Fp1, Fp2, F3, F4, C3, C4, P3, P4, O1, O2, F7, F8, T7, T8, P7, P8, FT9, FT10, P9, P10, PO9, PO10, F9, F10, F9′, F10′, TP9 and TP10. Cz served as initial common reference, and a forehead electrode (Afz) served as ground. Impedances were kept below 10 kΩ and were typically below 5 kΩ. The horizontal electro-oculogram (EOG) was recorded from F9′ to F10′ at the outer canthi of both eyes. The vertical EOG was monitored bipolarly from electrodes above and below the right eye. All signals were recorded with AC (0.05 Hz high pass, 40 Hz low pass, −6 dB attenuation, 12 dB/octave), and sampled at a rate of 250 Hz. Offline, ocular artefacts were corrected using Besa^TM^, Version 7.1. We analyzed visual ERPs relative to faces and auditory ERPs relative to voices paired with faces in the learning phases, and auditory ERPs relative to voices in the test phases. Note that in the learning phases, faces were presented before and during the presentation of to-be-learned voices, so that any early effects of facial distinctiveness could be assessed during visual stimulation alone. To this end, we segmented the learning phase EEG relative to face stimulus onset, from −200 to 1500 ms for faces, and from −200 to 2000 ms for voices. For faces, the 200 ms prior to face onset served as baseline; for voices, the 200 ms prior to voice onset (i.e., 1300 ms post face onset) served as baseline. For voices in the test phases, epochs were generated from −200 to 2000 ms relative to voice stimulus onset, with the 200 ms pre-voice onset interval serving as baseline. We used different epoch durations depending on stimulus type, because of different stimulus durations for faces and voices. Note, however, that voice epochs ended 2000 ms post-voice onset. This was because we did not plan to analyze time intervals beyond 2000 ms post-stimulus onset. In addition, an inclusion of later time-intervals that were not used for analysis might have resulted in unnecessary trial loss due to potential artifacts in these segments. Epochs contaminated by non-ocular artefacts were rejected from further analysis, with an amplitude threshold of 100 µV and a gradient criterion rejecting all epochs differing by more than 75 µV between consecutive data points. Any remaining artefacts of non-ocular origin were removed after visual inspection. In the test phases, only trials with correct responses (hits, correct rejections) were entered in the analysis. Continuous data were filtered with a 30 Hz, 12 db/oct zero-phase-shift low-pass filter prior to averaging. Trials were averaged separately for each channel and experimental condition, and collapsed across all learning phases and testing phases, respectively, in order to increase the signal-to-noise ratio. The average numbers of artefact-free trials per condition for faces in the learning phases were 34.1 and 33.8 (for distinctive vs. non-distinctive faces, respectively), with a minimum of 29 trials per condition and participant. The average numbers of artefact-free trials per condition for voices in the learning phase were 33.3 and 32.6 (for voices learned with distinctive vs. non-distinctive faces, respectively), with a minimum of 23 trials per condition and participant. The average numbers of correct and artefact-free trials per condition in the test phases were 23.3, 22.4, and 32.9 (for test voices learned with distinctive vs. non-distinctive faces, and novel voices, respectively), with a minimum of 13 trials per condition and participant. Each averaged ERP was recalculated to average reference, excluding the vertical and horizontal EOG channels.

**Figure 2:**
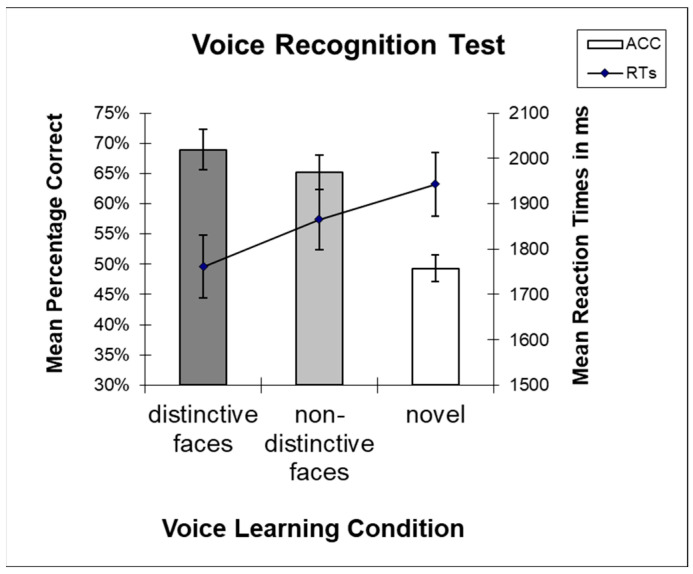
Mean ACCs (bars) and RTs (lines) for learning conditions in the voice recognition test with standard errors of the mean (SEM). Note the faster RTs for voices originally learned with distinctive (vs. non-distinctive) faces.

ERPs were quantified by mean amplitudes, determined separately for voices and faces, all relative to a 200 ms pre-stimulus baseline. For faces in the learning phases, we computed mean amplitudes for P100 (122–142 ms), N170 (152–182 ms), P200 (238–268 ms), N250 (280–380 ms), the LPC (500–700 ms), and sustained activity (700–1400 ms). P100 amplitude was quantified at O1 and O2, the LPC was measured at Cz and Pz, and the other face-elicited ERPs were measured at P9/P10 and PO9/PO10, respectively. For voices in the learning phases, we computed mean amplitudes for N1 (120–140 ms) and P2 (210–240 ms) at Cz, respectively, and for SA (650–1600 ms) at occipito–temporal sites P9/P10 and PO9/PO10, and at fronto-central electrodes (F3, F4, F7, F8, Fz, FP1, FP2), respectively. For voices in the test phases, we computed mean amplitudes for N1 (121–141 ms) and P2 (213–243 ms) at Cz, respectively, and for the “N250” (280–380 ms) as well as sustained activity (650–1600 ms) at occipito–temporal sites P9/P10 and PO9/PO10, and at fronto-central electrodes (F3, F4, F7, F8, Fz, FP1, FP2), respectively. Note, that while responses could be entered during test voice presentation, mean response times of around 1900 ms from voice onset (cf. Figure 2) suggest that any interference of motor responses with ERPs should be minimal.

Electrodes of interest for visual P100, N170, P200, N250, LPC and SA, as well as the auditory N1, P2 and “N250” were chosen a priori based on previous research [3,11,38,40,49,53,54,59,60], and confirmed by visual inspection. After narrowing down typical electrode sites and time windows for the above components based on this research, we used an automated search for minimal and maximal amplitudes to identify peak latencies of distinct peaks from the grand mean waveforms. This was performed separately for the learning and test phases, and for faces and voices, but averaged across learning conditions. Based on previous literature and visual inspection, we then determined time-windows around these peaks and calculated mean amplitudes. As for SA, there was no distinctive peak; the time-interval used for calculating mean amplitudes was chosen based on previous research (see above) and visual inspection.

## 3. Results

### 3.1. Behavioral Results Test Phase

We excluded reaction times (RTs) < 200 ms (0.47% of test trials) and performed ANOVAs for accuracies (ACCs) and correct RTs, with repeated measures on learning condition (voices learned with distinctive faces, non-distinctive faces, or novel voices). Where appropriate, Epsilon corrections for heterogeneity of covariances were performed with the Huynh–Feldt method [61] throughout. For raw data, see supplemental materials on the open science platform (OSF): https://osf.io/fkpav/ (accessed on 3 April 2023). (In response to a reviewer request, we also report signal-detection parameters per [62], H. Stanislaw and N. Todorov, “Calculation of signal detection theory measures,” (*Behavior Research Methods Instruments & Computers,* vol. 31, no. 1, pp. 137–149, 1999 1999. [Online]. Available: WOS:000079304500021), although this analysis is, strictly speaking, invalid, as we cannot calculate false alarms separately for both learning conditions (voices learned with distinctive vs. non-distinctive faces). This is due to our design, with both learning conditions being intermingled at the test, such that false alarm rates (i.e., incorrect responses to novel voices) are identical for both learning conditions. Nevertheless, we calculated d’ and c based on condition-specific hit rates and a mutual false alarm rate. These descriptive analyses suggested that discrimination performance for learned test voices and novel voices was well above chance (d‘_dist_ = 0.56 and d‘_non-dist_ = 0.41), and that participants adopted a liberal response criterion for the task, i.e., they were biased towards “old” responses (c_dist_ = −0.30 and c_non-dist_ = −0.23). For the reasons stated above, we refrained from any statistical comparisons.

#### 3.1.1. Accuracies

We observed a main effect of learning condition (*F* [2,54] = 11.689, *p* < 0.001, ƞ_p_^2^ = 0.302), with numerically more correct responses after learning voices with distinctive vs. non-distinctive faces (*M_dist_* = 0.69, *SD* = 0.17; *M_non-dist_* = 0.65, *SD* = 0.15), and with lowest accuracies for novel voices (*M_nov_* = 0.49, *SD* = 0.12). However, planned comparisons between voices learned with distinctive vs. non-distinctive faces yielded no significant effect (*t*(27) = 1.126, *p* = 0.135, one-tailed (Figure 2)).

#### 3.1.2. Reaction Times

We observed a main effect of learning condition (*F* [2,54] = 8.159, *p* = 0.003, ƞ_p_^2^ = 0.232), with the fastest responses to voices learned with distinctive faces and slowest responses for novel voices (*M_nov_* = 1.943, *SD* = 372). Planned comparisons between learning conditions with faces yielded significantly faster responses for voices learned with distinctive faces (*M_dist_* = 1.762, *SD* = 370) compared to non-distinctive faces ((*M_non-dist_* = 1.866, *SD* = 350), *t*(27) = −3.442, *p* < 0.001, one-tailed (Figure 2)).

### 3.2. EEG Results

ERP mean amplitudes were analyzed separately for voices and faces, by means of ANOVAs and t-tests with repeated measurements on learning condition. Note that the factor ‘learning condition’ had two levels (distinctive vs. non-distinctive faces) in the learning phases and three levels (voices learned with distinctive faces, voices learned with non-distinctive faces, or novel voices) in the test phases. For the P100, N170, P200, N250, auditory “N250”and occipito–temporal SA, additional factors of hemisphere (left, right) and site (temporal P9/P10 vs. occipito–temporal PO9/PO10) were included. For the auditory fronto-central SA, the additional factor site (F3, F4, F7, F8, Fz, FP1, FP2) was included. For the LPC, the additional factor site was included (Cz, Pz). Note that for reasons of readability and stringency, significant main effects of site, hemisphere, or interactions of site with hemisphere, which are independent of the experimental variables of interest and merely reflect the topography of a component, will not be reported. For a detailed summary of all statistical results, see Tables S1–S3 in the supplemental materials on OSF: https://osf.io/fkpav/ (accessed on 3 April 2023).

#### 3.2.1. ERPs Learning Phases

##### Faces

P100. The analysis revealed no significant effects.

*N170.* We observed larger amplitudes for distinctive vs. non-distinctive faces (*F* [1,27] = 14.203, *p* < 0.001, ƞ_p_^2^ = 0.345, (*M* = −1.8 µV and *M* = −1.2 µV, respectively)). No interactions of learning condition with site or hemisphere were observed (all *Fs* < 1.01).

P200. There were smaller amplitudes (i.e., less positivity/more negativity) for distinctive vs. non-distinctive faces (*F* [1,27] = 6.666, *p* = 0.016, ƞ_p_^2^ = 0.198 (*M* = 0.43 µV and *M* = 0.94 µV, respectively)). No interactions of learning condition with site or hemisphere were significant (all *Fs* [1,27] ≤ 1.764; 0.195 ≤ *ps* ≥ 0.908).

N250. We observed larger (i.e., more negative) amplitudes for distinctive vs. non-distinctive faces (*F* [1,27] = 12.840, *p* < 0.001, ƞ_p_^2^ = 0.322 (*M* = −0.54 µV and *M* = 0.12 µV, respectively)). No interactions of learning condition with site or hemisphere were observed (all *Fs*(1, 27) ≤ 1.572; 0. 221≤ *ps* ≥ 0.964).

LPC. The analysis showed larger positive amplitudes for distinctive vs. non-distinctive faces (*F* [1,27] = 32.545, *p* < 0.001, ƞ_p_^2^ = 0.547 (*M* = 3.8 µV and *M* = 2.3 µV, respectively)), without an interaction between learning condition and site (F < 1).

SA (700–1400 ms). We observed more negative amplitudes for distinctive vs. non-distinctive faces (*F* [1,27] = 14.808, *p* < 0.001, ƞ_p_^2^ = 0.354 (*M* = −3.2 µV and *M* = −2.4 µV, respectively)). No interactions of learning condition with site or hemisphere were observed (all *Fs* [1,27] ≤ 2.617; 0.117 ≤ *ps* ≥ 0.926).

##### Voices

N1 and P2. There were no significant effects.

**Figure 3:**
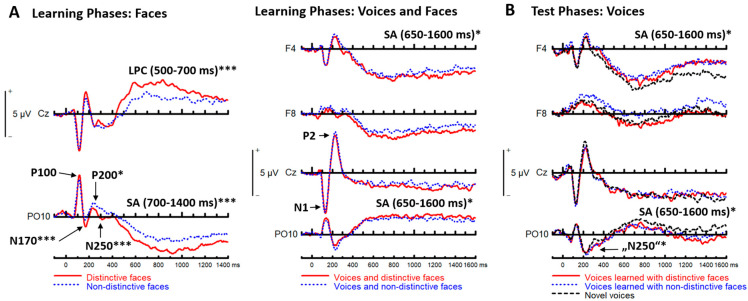
Grand mean averages of uni−modal face-elicited ERPs at Cz and PO10 for both learning conditions in the learning phases (**A**, **left**), voice−elicited ERPs at F4, F8, Cz and PO10 for voices presented with distinctive or non−distinctive faces (**A**, **right**), unimodal voice−elicited ERPs at F4, F8, Cz and PO10 in the test phases, for voices that had been originally learned with distinctive or non-distinctive faces, and for novel voices (**B**) (* *p* < 0.05, *** *p* < 0.001).

SA (650–1600 ms). The analysis at occipito–temporal P9/P10 and PO9/PO10 revealed more positive amplitudes for voices accompanied by distinctive faces vs. voices accompanied by non-distinctive faces (*F* [1,27] = 4.939, *p* = 0.035, ƞ_p_^2^ = 0.155 (*M* = 2.3 µV and *M* = 1.9 µV, respectively; cf. Figure 3, Panel A, bottom right)). No interactions of learning condition with site or hemisphere were observed (all *Fs* < 1). The ANOVA at fronto-central sites revealed more negative amplitudes for the distinctive compared to the non-distinctive condition (*F* [1,27] = 6.685, *p* = 0.015, ƞ_p_^2^ = 0.198 (*M* = −1.5 µV and *M* = −1.2 µV, respectively)). There was no interaction with site (*F* [6,162] = 1.020, *p* = 0.415). For grand mean averages of learning and test-phase ERPs at electrodes of interest, see Figure 3. For an overview of all electrode sites, see supplemental materials (Figures S1–S3).

#### 3.2.2. ERPs Test Phases (Voices)

N1 and P2. There were no significant effects.

“N250”. We obtained a main effect of learning condition (*F* [2,54] = 3.357, *p* = 0.042, ƞ_p_^2^ = 0.111), with no interactions of learning condition with site or hemisphere (all *Fs* < 1). The effect of the learning condition reflected larger amplitudes for learned voices compared to novel voices, as revealed by pairwise comparisons of voices learned with distinctive faces vs. novel voices (*p* = 0.027), and voices learned with non-distinctive faces vs. novel voices (*p* = 0.024), as well as no significant difference between voices learned with distinctive vs. non-distinctive faces (*p* = 0.835).

**Figure 4:**
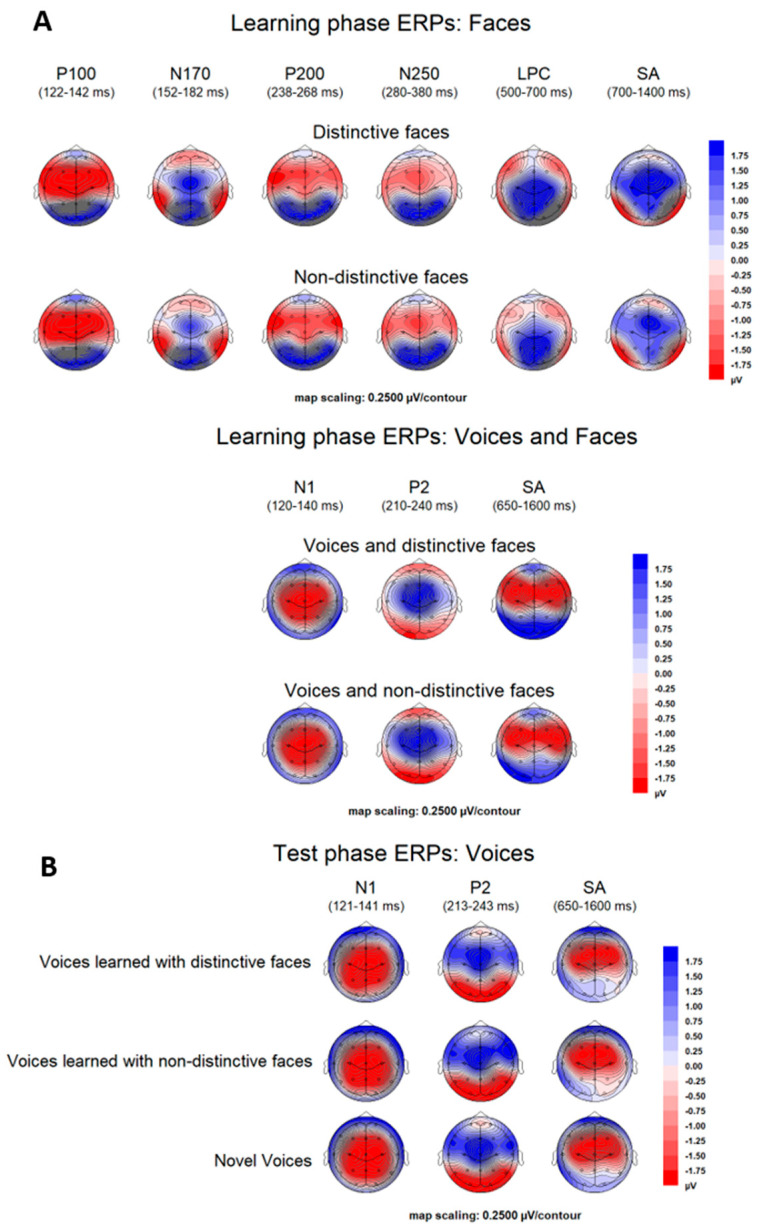
Scalp topographical voltage maps (topview, spherical spline interpolation, 110° equidistant projection) of ERPs in learning phases (**A**)**,** as elicited by distinctive and non−distinctive faces (**A**, **top**), or by voices combined with distinctive or non−distinctive faces (**A**, **bottom**), and ERPs in test phases (**B**)**,** as elicited by unimodal voices that had been originally learned with distinctive and non−distinctive faces.

*SA* (650–1600 ms). At occipito–temporal sites we observed a main effect of learning condition (*F* [2,54] = 4.110, *p* = 0.022, ƞ_p_^2^ = 0.132), with no interactions of learning condition with site or hemisphere (all *Fs* < 1). The effect of learning condition again reflected larger amplitudes for learned voices compared to novel voices, as revealed by comparisons of voices learned with distinctive faces vs. novel voices (*p* = 0.037), and voices learned with non-distinctive faces vs. novel voices (*p* = 0.009), as well as no significant difference between voices learned with distinctive vs. non-distinctive faces (*p* = 0.945) (cf. Figure 3, Panel B, bottom). At fronto-central electrodes, there was no main effect for learning condition (*F* [2,54] = 2.241, *p* = 0.116, ƞ_p_^2^ = 0.077), but a two-way interaction between learning condition and site (*F* [12,324] = 2.051, *p* = 0.037, ƞ_p_^2^ = 0.071). The interaction was followed up by separate ANOVAs with the factor ‘learning condition’ for each electrode. We observed an effect of learning condition at F4, (*F* [2,54] = 4.709, *p* = 0.013, ƞ_p_^2^ = 0.149). Pairwise comparisons showed less negativity for voices learned with non-distinctive faces vs. novel voices (*p* = 0.002), and a similar trend for voices learned with distinctive faces vs. novel voices (*p* = 0.055), as well as no significant difference between voices learned with distinctive vs. non-distinctive faces (*p* = 0.661). At F8, we observed a main effect for learning condition (*F* [2,54] = 6.153, *p* = 0.004, ƞ_p_^2^ = 0.186). According to pair-wise comparisons, this was due to more positive amplitudes for voices learned with non-distinctive faces vs. novel voices (*p* = 0.008) and vs. voices learned with distinctive faces (*p* = 0.004), which did not differ from novel voices (*p* = 0.371). For Fz, there was a trend for learning condition (*F* [2,54] = 2.516, *p* = 0.090, ƞ_p_^2^ = 0.085; for all other electrodes *Fs* < 1). Voltage maps for the learning and test phase ERPs can be found in Figure 4.

#### 3.2.3. Source Localization Test Phase

**Figure 5:**
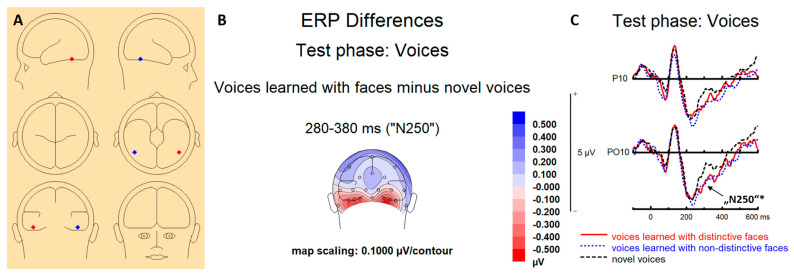
(**A**) Source localization for the “N250” to test voices (difference between voices learned with faces vs. novel voices). (**B**) Topographical voltage map of the same difference (spherical spline interpolation, 110° equidistant projection, backview) for the N250 time window. (**C**) Grand mean averages of uni-modal voice−elicited “N250” at P10 and PO10 in the test phases, for voices that had been learned with distinctive or non−distinctive faces, and for novel voices (* *p* < 0.05).

To locate the effect of learned voices (in the present situation, when voices were combined with a face during learning) on test voices (when these were presented in the absence of a face), we performed an exploratory source localization (Besa^TM^ 7.1). We opted for analyzing the early portion of the N250 time window (282–318 ms), in which a principal components analysis (PCA) suggested that the first two components explained a maximum of data variance (82.6% and 13.9%, respectively). A four-shell-ellipsoidal model with two symmetrical regional sources suggested a source in the fusiform gyrus (Talairach-coordinates: x = ±46, y = −58, z = −21) for the difference between audio-visually learned voices and novel voices (see Figure 5).

## 4. Discussion

In the learning phases, our finding of larger negativity in the time range of the N170, P200, and N250 in response to distinctive vs. non-distinctive faces confirms an extensive set of previous findings, which collectively indicates that distinctive faces facilitate the formation of face representations [3,26,34,43,63]. Similarly, findings of a larger LPC relative to distinctive vs. non-distinctive faces also replicate reports in the above papers. In addition, we also identified an enhanced and sustained occipito–temporal negativity related to distinctive faces in the learning phases. This finding could be tentatively interpreted as an indicator of superior encoding of distinctive faces. Specifically, larger occipito–temporal negativity has been previously described in the context of a face’s encoding into memory [51], in the context of familiar face recognition [52], and also as a correlate of familiar face distinctiveness in terms of the distance to a norm [36].

For voices that were presented with distinctive or non-distinctive faces during learning, we observed no evidence for a modulation of early auditory ERPs (N1, P2) in the learning phases, even though distinctive faces were shown 1500 ms before voice onset and remained on-screen during voice presentation. Accordingly, we see no evidence from early auditory-evoked potentials that facial distinctiveness induced differences in rapid attentional processing of the voice (e.g., via sensory gating). This could have been a possibility, especially because the auditory N1 is known to be highly sensitive to selective attention, with larger amplitudes reflecting higher degrees of selective attention [44,64]. In fact, one other study investigated cross-modal (face-to-voice) identity priming [50], and found that information conveyed by the face can modulate sensory processing of a subsequent voice in terms of the amplitude of the N1 component. Various differences between studies (including experimental design, stimuli, or learning conditions) may account for discrepancies between these findings and the present results. Similarly, one might have expected a modulation in the P2 time range in the present study, based on magnetoencephalographic (MEG) data [65]. Specifically, this study suggested that the temporal auditory field response M200 to learned voices is facilitated by prior audio-visual voice–face learning compared to an audio-visual control condition (voice–occupation learning). The authors speculated that their M200 effect reflected facilitated extraction of the spectral composition of learned voices following voice–face learning. Based on these findings, the absence of face distinctiveness effects in the present P2 allows for at least three interpretations: (i) facial distinctiveness during voice–face learning does not modulate (spectral) low-level analysis in subsequent learned voices, (ii) although facial distinctiveness can modulate low-level auditory voice analysis, the present manipulation was too weak to elicit a measurable effect in the P2, or (iii) any other difference between studies may account for the discrepant results.

Despite the absence of facial distinctiveness effects on early voice-elicited ERPs, we did observe differences between ERPs for voices paired with distinctive or typical faces for the later sustained activity time-interval. At occipito–temporal sites, mean amplitudes were more positive, and at frontal sites they were more negative, for voices paired with distinctive faces. Although it is difficult to directly relate these findings to published results, they may be reminiscent of recent findings that showed sustained positive ERPs relative to familiar voices [66]. One can imagine several mechanisms by which facial distinctiveness could affect encoding of a voice. For instance, distinctive faces might cause enhanced sustained attention and better encoding relative to a simultaneously presented voice, perhaps because the information provided by two modalities is usually redundant, at least to some extent (but see also [29], e.g., [67]). Alternatively, by capturing attention, distinctive faces might impede voice encoding and result in costs in terms of an enhanced face-overshadowing effect (e.g., [16,20]).

In the test phases of the present study, the speed of voice recognition suggests benefits rather than costs of distinctive faces during voice encoding: Voices that had been initially learned together with distinctive faces were recognized more efficiently at the test, compared to voices that had been initially learned with typical faces. This advantage was significant in response times but not in accuracy. Nevertheless, accuracy was numerically higher for voices learned with distinctive faces, which allows us to exclude the possibility that this benefit in RTs simply reflected differences in speed–accuracy trade-off. However, the precise mechanisms by which distinctive faces modulate voice encoding clearly require further specification. In parallel, the interpretation of the sustained positivity relative to voices paired with distinctive faces remains speculative at present and warrants further investigation. We suggest that face distinctiveness may facilitate the acquisition of multimodal person representations during learning, as well as the subsequent access to these representations at the test. The frontal effect during learning might also point to an additional strategic component during encoding. Faster responses to voices learned with distinctive vs. non-distinctive faces may therefore reflect a more efficient processing of learned voices.

An Intriguing finding in the ERPs at the test was that, even though no faces were present, unimodal test voices induced an N250-like response, as well as a sustained negativity at occipito–temporal sites, and sustained positivity at frontal sites. Remarkably, in particular, the “N250” response exhibited a prominent occipito–temporal scalp topography, and is reminiscent of ERP phenomena that were previously reported in the context of face memory (e.g., [36,51,52]). It is important to note that the N250 and sustained activity at the test were independent of face distinctiveness during learning (apart from the local effect at F8), and therefore this represents a contrast between learned and novel voices under the audio-visual learning conditions of the present study. Although a direct comparison is difficult, similar occipito–temporal differences between learned and novel voices were not evident in earlier auditory-only ERP research on voice learning and recognition [11,68]. Moreover, fMRI research into the neural correlates of auditory-only learning and recognition of voices did not suggest a contribution of occipito–temporal cortex areas. Rather, this research consistently suggested that areas in the vicinity of the superior temporal sulcus (STS) and the inferior frontal gyrus (IFG) may be the most relevant areas for voice learning and recognition in those unimodal situations [69,70]. The only difference at the test between ERPs for voices learned with distinctive faces compared to voices learned with non-distinctive faces was the observation of increased positivity during the SA time interval at the frontal electrode F8. In a recent ERP study on intentional learning and forgetting of voices [12], there was a similar right frontal effect for test voices that had been prompted to be forgotten during learning vs. voices that had been prompted to be remembered for a subsequent test. The finding was tentatively interpreted to reflect increased effort, general decision-making and monitoring processes for intentionally forgotten voices, potentially stemming from activity in the right dorsolateral prefrontal cortex (see also [71,72,73]). Similarly, the present distinctiveness effect in the SA time interval at F8 may tentatively reflect increased retrieval effort for test voices that had been learned with non-distinctive as compared to distinctive faces.

An N250-like ERP has not been previously associated with voice memory, although one recent auditory-only ERP study on neural correlates of voice recognition [66] has identified an enhanced and broadly distributed “N250” (300–350 ms) response to trained as compared to unknown voices. Remarkably, an inspection of the present scalp topography of the N250 difference between (audio-visually) learned and novel voices, as well as exploratory dipole source localization of the early portion of this difference was consistent with the idea that the fusiform gyrus in the ventral occipito–temporal cortex contributed to voice recognition in the present study. We should make clear that great caution is indicated when interpreting source localization from scalp-recorded ERPs alone. At the same time, the fusiform gyrus is a key area in the face processing network [74,75]. Moreover, findings that the fusiform gyrus contributes to the face-elicited N250 response [60] are quite consistent with converging evidence existing from both magnetoencephalography (MEG; [76]) and fMRI (for review, see [35,77]).

Interestingly, MEG research had suggested a remarkably early voice-elicited activation (~110 ms) of the posterior fusiform gyrus, when voices had been learned with concurrent talking faces vs. images of occupations [65]. This finding was accompanied by a behavioral ‘face-benefit’, with higher voice recognition rates after voice–face learning, compared to voice–occupation learning. Schall et al. interpreted their MEG effect with a direct spread of activation from early auditory areas to face-sensitive areas, in line with fMRI findings of a functional connection [2,78] and direct structural connections [7] between temporal voice areas and the FFA. The present voice-elicited N250-like ERP may add to this evidence for an early activation of face areas by voices, once speaker representations have been established via audio-visual learning.

While the source localization of the present voice-induced N250 in fusiform gyrus in young healthy adults potentially represents a novel finding for scalp-recorded EEG, it may be noted that our findings can be related to a very recent study using intracranial EEG recordings from epilepsy patients, in which the authors directly demonstrated an electrophysiological response in fusiform cortex in response to famous voices, with onset latencies close to 300 ms [79]. Although more research is necessary to delineate in more detail the contribution of fusiform cortex to voice learning, the present findings support the notion of an early perceptual interaction in areas implicated in voice and face processing [2,7] (see also [80]). Note that such an early interaction does not exclude the possibility of an additional later, post-perceptual crosstalk between voice and face processing pathways, as suggested by influential models (e.g., [1,81]).

It may be relevant to consider evidence from another domain of research that investigates disorders of person recognition: substantial impairments to the recognition of faces (prosopagnosia) or voices (phonagnosia) can be acquired as a result of brain damage (e.g., [82,83]); even in the absence of structural brain changes, such impairments can occur in a “developmental” or “congenital” variant [84,85]. Voice recognition abilities are typically preserved in cases with both developmental [86,87] and acquired prosopagnosia [88], thus suggesting that disorders in face and voice recognition are dissociable. At the same time, multimodal deficits in recognizing people from both faces and voices have also been reported. Such multimodal deficits have been variably interpreted as potentially being caused by unilateral right-hemispheric anterior temporal lesions [89,90,91], or as being related to bilateral lesions [88,92]. In the present study, the N250 and sustained activity for learned vs. novel voices in the test phase did not exhibit significant lateralization to the right hemisphere.

Several limitations should be noted while interpreting the present results. First, we note that voices were combined with static faces during learning. Based on studies of audio-visual integration in the recognition of familiar voices (e.g., [24,93]), it seems possible that an ecologically more valid design in which voices would be presented together with time-synchronized speaking faces (rather than static faces) during learning might elicit even stronger effects of facial distinctiveness on voice learning. Nevertheless, evidence for face-voice integration in early ERP responses in the context of person identification has been reported even for combinations with static faces (e.g., [94]). Moreover, it may be noted that voice learning performance in the present study was only moderate overall, despite repetition of study voices in six learning phases. The degree to which audio-visual benefits to voice learning and the latter’s potential modulation by facial distinctiveness develop over time remains to be better understood (e.g., [13]).

## 5. Conclusions

Overall, the present study used ERP recordings in the context of a voice learning task in which voices were learned in combination with distinctive or typical faces, and in which voice memory was tested with only auditory information. The present results indicate that the combination with distinctive faces during voice learning can lead to faster and more efficient auditory-only voice recognition at the test. Moreover, voices previously learned with faces elicited occipito–temporal negative ERPs at the test, both in an early N250 (280–380 ms) and in a sustained negativity (650–1600 ms). At fronto-central sites, learned voices evoked a sustained positivity (650–1600 ms) compared to novel voices. In the later time-range there was also some evidence for increased retrieval effort for voices that had been initially learned with non-distinctive faces. Both topography and an exploratory source localization of the N250 effect were similar to previous effects seen in the context of face recognition and were consistent with a source in the fusiform gyrus. Overall, while more research is needed to delineate the mechanisms of audio-visual effects on voice learning, the present study contributes to recent research that suggests an early interaction between voice and face processing areas to encode and activate multimodal person-representations in memory.

## Data Availability

The data presented in this study are openly available on the Open Science Framework (OSF): https://osf.io/fkpav/ (accessed on 3 April 2023).

## Supplementary Materials

The following supporting information can be downloaded at OSF: https://osf.io/fkpav/ (accessed on 3 April 2023). Table S1: Summary of statistical analyses (F-values, t-values, dfs in parentheses, and effect sizes) of ERP mean amplitudes to faces in the learning phases; Table S2: Summary of statistical analyses (F-values, t-values, dfs in parentheses, and effect sizes) of ERP mean amplitudes to voices and faces in the learning phases; Table S3: Summary of statistical analyses (F-values, dfs in parentheses, and effect sizes) of ERP mean amplitudes to voices in the test phases; Figure S1: Grand mean averages of voice-elicited ERPs in learning phases, shown separately for learning conditions (concurrent distinctive or non-distinctive faces) at all 30 electrode sites; Figure S2: Grand mean averages of voice-elicited ERPs in test phases, shown separately for learning conditions (voices learned with distinctive or non-distinctive faces, novel voices) at all 30 electrode sites; Figure S3: Grand mean averages of voice-elicited ERPs in test phases, shown separately for learning conditions (voices learned with distinctive or non-distinctive faces, novel voices) at all 30 electrode sites.

## References

[1] Young A.W., Frühholz S., Schweinberger S.R. (2020). Face and Voice Perception: Understanding Commonalities and Differences. Trends Cogn. Sci..

[2] von Kriegstein K., Kleinschmidt A., Sterzer P., Giraud A.L. (2005). Interaction of face and voice areas during speaker recognition. J. Cogn. Neurosci..

[3] Kaufmann J.M., Schweinberger S.R. (2012). The faces you remember: Caricaturing shape facilitates brain processes reflecting the acquisition of new face representations. Biol. Psychol..

[4] Skuk V.G., Schweinberger S.R. (2013). Gender differences in familiar voice identification. Hear. Res..

[5] Blank H., Wieland N., von Kriegstein K. (2014). Person recognition and the brain: Merging evidence from patients and healthy individuals. Neurosci. Biobehav. Rev..

[6] von Kriegstein K., Dogan O., Grüter M., Giraud A.-L., Kell C.A., Grüter T., Kleinschmidt A., Kiebel S.J. (2008). Simulation of talking faces in the human brain improves auditory speech recognition. Proc. Natl. Acad. Sci. USA.

[7] Blank H., Anwander A., von Kriegstein K. (2011). Direct Structural Connections between Voice- and Face-Recognition Areas. J. Neurosci. Off. J. Soc. Neurosci..

[8] Ellis H.D., Jones D.M., Mosdell N. (1997). Intra- and inter-modal repetition priming of familiar faces and voices. Br. J. Psychol..

[9] Lavan N., Collins M.R.N., Miah J.F.M. (2022). Audiovisual identity perception from naturally-varying stimuli is driven by visual information. Br. J. Psychol..

[10] Hanley J.R., Turner J.M. (2000). Why are familiar-only experiences more frequent for voices than for faces?. Q. J. Exp. Psychol. Sect. A-Hum. Exp. Psychol..

[11] Zäske R., Volberg G., Kovacs G., Schweinberger S.R. (2014). Electrophysiological Correlates of Voice Learning and Recognition. J. Neurosci..

[12] Humble D., Schweinberger S.R., Dobel C., Zäske R. (2019). Voices to remember: Comparing neural signatures of intentional and non-intentional voice learning and recognition. Brain Res..

[13] Zäske R., Mühl C., Schweinberger S.R. (2015). Benefits for Voice Learning Caused by Concurrent Faces Develop over Time. PLoS ONE.

[14] Humble D., Schweinberger S.R., Mayer A., Jesgarzewsky T.L., Dobel C., Zäske R. (2022). The Jena Voice Learning and Memory Test (JVLMT): A standardized tool for assessing the ability to learn and recognize voices. Behav. Res. Methods.

[15] Sheffert S.M., Olson E. (2004). Audiovisual speech facilitates voice learning. Percept. Psychophys..

[16] Tomlin R.J., Stevenage S.V., Hammond S. (2017). Putting the pieces together: Revealing face-voice integration through the facial overshadowing effect. Vis. Cogn..

[17] Cook S., Wilding J. (1997). Earwitness testimony 2. Voices, faces and context. Appl. Cogn. Psychol..

[18] McAllister H.A., Dale R.H.I., Bregman N.J., McCabe A., Cotton C.R. (1993). When Eyewitnesses Are Also Earwitnesses—Effects on Visual and Voice Identifications. Basic Appl. Soc. Psychol..

[19] Stevenage S.V., Howland A., Tippelt A. (2011). Interference in Eyewitness and Earwitness Recognition. Appl. Cogn. Psychol..

[20] Lavan N., Bamaniya N.R., Muse M.M., Price R.L.M., Mareschal I. (2023). The effects of the presence of a face and direct eye gaze on voice identity learning. Br. J. Psychol..

[21] Cook S., Wilding J. (2001). Earwitness testimony: Effects of exposure and attention on the face overshadowing effect. Br. J. Psychol..

[22] Schweinberger S.R., Robertson D.M.C. (2017). Audiovisual integration in familiar person recognition. Vis. Cogn..

[23] Robertson D.M.C., Schweinberger S.R. (2010). The role of audiovisual asynchrony in person recognition. Q. J. Exp. Psychol..

[24] Schweinberger S.R., Robertson D., Kaufmann R.M. (2007). Hearing facial identities. Q. J. Exp. Psychol..

[25] Latinus M., VanRullen R., Taylor M.J. (2010). Top-down and bottom-up modulation in processing bimodal face/voice stimuli. BMC Neurosci..

[26] Schulz C., Kaufmann J.M., Kurt A., Schweinberger S.R. (2012). Faces forming traces: Neurophysiological correlates of learning naturally distinctive and caricatured faces. Neuroimage.

[27] Mullennix J., Ross A., Smith C., Kuykendall K., Conard J., Barb S. (2011). Typicality Effects on Memory for Voice: Implications for Earwitness Testimony. Appl. Cogn. Psychol..

[28] Stevenage S.V., Neil G.J., Parsons B., Humphreys A. (2018). A sound effect: Exploration of the distinctiveness advantage in voice recognition. Appl. Cogn. Psychol..

[29] Zäske R., Skuk V.G., Schweinberger S.R. (2020). Attractiveness and distinctiveness between speakers’ voices in naturalistic speech and their faces are uncorrelated. R. Soc. Open Sci..

[30] Karlsson T., Schaefer H., Barton J.J.S., Corrow S.L. (2023). Effects of Voice and Biographic Data on Face Encoding. Brain Sci..

[31] Stevenage S.V., Hamlin I., Ford B. (2017). Distinctiveness helps when matching static faces and voices. J. Cogn. Psychol..

[32] Bülthoff I., Newell F.N. (2015). Distinctive voices enhance the visual recognition of unfamiliar faces. Cognition.

[33] Bülthoff I., Newell F.N. (2017). Crossmodal priming of unfamiliar faces supports early interactions between voices and faces in person perception. Vis. Cogn..

[34] Zheng X., Mondloch C.J., Segalowitz S.J. (2012). The timing of individual face recognition in the brain. Neuropsychologia.

[35] Schweinberger S.R., Neumann M.F. (2016). Repetition effects in human ERPs to faces. Cortex.

[36] Wuttke S.J., Schweinberger S.R. (2019). The P200 predominantly reflects distance-to-norm in face space whereas the N250 reflects activation of identity-specific representations of known faces. Biol. Psychol..

[37] Amihai I., Deouell L.Y., Bentin S. (2011). Neural adaptation is related to face repetition irrespective of identity: A reappraisal of the N170 effect. Exp. Brain Res..

[38] Eimer M., Calder A.J., Rhodes G., Johnson M.H., Haxby J.V. (2011). The face-sensitive N170 component of the event-related brain potential. The Oxford Handbook of Face Perception.

[39] Eimer M., Gosling A., Duchaine B. (2012). Electrophysiological markers of covert face recognition in developmental prosopagnosia. Brain.

[40] Schulz C., Kaufmann J.M., Walther L., Schweinberger S.R. (2012). Effects of anticaricaturing vs. caricaturing and their neural correlates elucidate a role of shape for face learning. Neuropsychologia.

[41] Hillyard S.A., Vogel E.K., Luck S.J. (1998). Sensory gain control (amplification) as a mechanism of selective attention: Electrophysiological and neuroimaging evidence. Philos. Trans. R. Soc. Lond. Ser. B-Biol. Sci..

[42] Itz M.L., Schweinberger S.R., Kaufmann J.M. (2016). Effects of Caricaturing in Shape or Color on Familiarity Decisions for Familiar and Unfamiliar Faces. PLoS ONE.

[43] Itz M.L., Schweinberger S.R., Schulz C., Kaufmann J.M. (2014). Neural correlates of facilitations in face learning by selective caricaturing of facial shape or reflectance. Neuroimage.

[44] Hillyard S.A., Hink R.F., Schwent V.L., Picton T.W. (1973). Electrical Signs of Selective Attention in Human Brain. Science.

[45] Altmann C.F., Nakata H., Noguchi Y., Inui K., Hoshiyama M., Kaneoke Y., Kakigi R. (2008). Temporal dynamics of adaptation to natural sounds in the human auditory cortex. Cereb. Cortex.

[46] Kuriki S., Ohta K., Koyama S. (2007). Persistent responsiveness of long-latency auditory cortical activities in response to repeated stimuli of musical timbre and vowel sounds. Cereb. Cortex.

[47] Nussbaum C., Schirmer A., Schweinberger S.R. (2022). Contributions of fundamental frequency and timbre to vocal emotion perception and their electrophysiological correlates. Soc. Cogn. Affect. Neurosci..

[48] Schirmer A., Kotz S.A. (2006). Beyond the right hemisphere: Brain mechanisms mediating vocal emotional processing. Trends Cogn. Sci..

[49] Zäske R., Schweinberger S.R., Kaufmann J.M., Kawahara H. (2009). In the ear of the beholder: Neural correlates of adaptation to voice gender. Eur. J. Neurosci..

[50] Föcker J., Hölig C., Best A., Roeder B. (2011). Crossmodal interaction of facial and vocal person identity information: An event-related potential study. Brain Res..

[51] Sommer W., Heinz A., Leuthold H., Matt J., Schweinberger S.R. (1995). Metamemory, Distinctiveness, and Event-Related Potentials in Recognition Memory for Faces. Mem. Cogn..

[52] Wiese H., Tüttenberg S.C., Ingram B., Chan C.Y.X., Gurbuz Z., Burton A.M., Young A.W. (2019). A Robust Neural Index of High Face Familiarity. Psychol. Sci..

[53] Schweinberger S.R. (2001). Human brain potential correlates of voice priming and voice recognition. Neuropsychologia.

[54] Picton T.W., Woods D.L., Proulx G.B. (1978). Human Auditory Sustained Potentials 1. Nature of Response. Electroencephalogr. Clin. Neurophysiol..

[55] Faul F., Erdfelder E., Lang A.G., Buchner A. (2007). G*Power 3: A flexible statistical power analysis program for the social, behavioral, and biomedical sciences. Behav. Res. Methods.

[56] Burton A.M., White D., McNeill A. (2010). The Glasgow Face Matching Test. Behav. Res. Methods.

[57] Phillips P.J., Wechsler H., Huang J., Rauss P.J. (1998). The FERET database and evaluation procedure for face-recognition algorithms. Image Vis. Comput..

[58] Phillips P.J., Moon H., Rizvi S.A., Rauss P.J. (2000). The FERET evaluation methodology for face-recognition algorithms. IEEE Trans. Pattern Anal. Mach. Intell..

[59] Kaufmann J.M., Schweinberger S.R., Burton A. (2009). N250 ERP Correlates of the Acquisition of Face Representations across Different Images. J. Cogn. Neurosci..

[60] Schweinberger S.R., Pickering E.C., Jentzsch I., Burton A.M., Kaufmann J.M. (2002). Event-related brain potential evidence for a response of inferior temporal cortex to familiar face repetitions. Cogn. Brain Res..

[61] Huynh H., Feldt L.S. (1976). Estimation of the box correction for degrees of freedom from sample data in randomized block and split block designs. J. Educ. Stat..

[62] Stanislaw H., Todorov N. (1999). Calculation of signal detection theory measures. Behav. Res. Methods Instrum. Comput..

[63] Itz M.L., Schweinberger S.R., Kaufmann J.M. (2017). Caricature generalization benefits for faces learned with enhanced idiosyncratic shape or texture. Cogn. Affect. Behav. Neurosci..

[64] Talsma D., Woldorff M.G. (2005). Selective attention and multisensory integration: Multiple phases of effects on the evoked brain activity. J. Cogn. Neurosci..

[65] Schall S., Kiebel S.J., Maess B., von Kriegstein K. (2013). Early auditory sensory processing of voices is facilitated by visual mechanisms. Neuroimage.

[66] Plante-Hebert J., Boucher V.J., Jemel B. (2021). The processing of intimately familiar and unfamiliar voices: Specific neural responses of speaker recognition and identification. PLoS ONE.

[67] Kamachi M., Hill H., Lander K., Vatikiotis-Bateson E. (2003). Putting the face to the voice’: Matching identity across modality. Curr. Biol..

[68] Zäske R., Limbach K., Schneider D., Skuk V.G., Dobel C., Guntinas-Lichius O., Schweinberger S.R. (2018). Electrophysiological correlates of voice memory for young and old speakers in young and old listeners. Neuropsychologia.

[69] Latinus M., Crabbe F., Belin P. (2011). Learning-Induced Changes in the Cerebral Processing of Voice Identity. Cereb. Cortex.

[70] Zäske R., Hasan B.A.S., Belin P. (2017). It doesn’t matter what you say: fMRI correlates of voice learning and recognition independent of speech content. Cortex.

[71] Rizio A.A., Dennis N.A. (2017). Recollection after inhibition: The effects of intentional forgetting on the neural correlates of retrieval. Cogn. Neurosci..

[72] Hayama H.R., Johnson J.D., Rugg M.D. (2008). The relationship between the right frontal old/new ERP effect and post-retrieval monitoring: Specific or non-specific?. Neuropsychologia.

[73] Hayama H.R., Rugg M.D. (2009). Right dorsolateral prefrontal cortex is engaged during post-retrieval processing of both episodic and semantic information. Neuropsychologia.

[74] Puce A., Allison T., Asgari M., Gore J.C., McCarthy G. (1996). Differential sensitivity of human visual cortex to faces, letterstrings, and textures: A functional magnetic resonance imaging study. J. Neurosci..

[75] Kanwisher N., McDermott J., Chun M.M. (1997). The fusiform face area: A module in human extrastriate cortex specialized for face perception. J. Neurosci..

[76] Schweinberger S.R., Kaufmann J.M., Moratti S., Keil A., Burton A.M. (2007). Brain responses to repetitions of human and animal faces, inverted faces, and objects—An MEG study. Brain Res..

[77] Eger E., Schweinberger S.R., Dolan R.J., Henson R.N. (2005). Familiarity enhances invariance of face representations in human ventral visual cortex: fMRI evidence. Neuroimage.

[78] von Kriegstein K., Giraud A.L. (2006). Implicit multisensory associations influence voice recognition. PLoS Biol..

[79] Rhone A.E., Rupp K., Hect J.L., Harford E.E., Tranel D., Howard M.A., Abel T.J. (2023). Electrocorticography reveals the dynamics of famous voice responses in human fusiform gyrus. J. Neurophysiol..

[80] Blank H., Kiebel S.J., von Kriegstein K. (2015). How the Human Brain Exchanges Information Across Sensory Modalities to Recognize Other People. Hum. Brain Mapp..

[81] Belin P., Fecteau S., Bedard C. (2004). Thinking the voice: Neural correlates of voice perception. Trends Cogn. Sci..

[82] Bodamer J. (1947). Prosopagnosie. Arch. Der Psychiatr. Nervenkrankh..

[83] van Lancker D.R., Kreiman J., Cummings J. (1989). Voice Perception Deficits—Neuroanatomical Correlates of Phonagnosia. J. Clin. Exp. Neuropsychol..

[84] Garrido L., Eisner F., McGettigan C., Stewart L., Sauter D., Hanley J.R., Schweinberger S.R., Warren J.D., Duchaine B. (2009). Developmental phonagnosia: A selective deficit of vocal identity recognition. Neuropsychologia.

[85] McConachie H.R. (1976). Developmental Prosopagnosia. A Single Case Report. Cortex.

[86] Liu R.R., Corrow S.L., Pancaroglu R., Duchaine B., Barton J.J.S. (2015). The processing of voice identity in developmental prosopagnosia. Cortex.

[87] Tsantani M., Cook R. (2020). Normal recognition of famous voices in developmental prosopagnosia. Sci. Rep..

[88] Liu R.R., Pancaroglu R., Hills C.S., Duchaine B., Barton J.J.S. (2016). Voice Recognition in Face-Blind Patients. Cereb. Cortex.

[89] Cosseddu M., Gazzina S., Borroni B., Padovani A., Gainotti G. (2018). Multimodal Face and Voice Recognition Disorders in a Case With Unilateral Right Anterior Temporal Lobe Atrophy. Neuropsychology.

[90] Gainotti G. (2013). Laterality effects in normal subjects’ recognition of familiar faces, voices and names. Perceptual and representational components. Neuropsychologia.

[91] Schroeger A., Kaufmann J.M., Zäske R., Kovacs G., Klos T., Schweinberger S.R. (2022). Atypical prosopagnosia following right hemispheric stroke: A 23-year follow-up study with MT. Cogn. Neuropsychol..

[92] Neuner F., Schweinberger S.R. (2000). Neuropsychological impairments in the recognition of faces, voices, and personal names. Brain Cogn..

[93] Schweinberger S.R., Kloth N., Robertson D.M. (2011). Hearing facial identities: Brain correlates of face-voice integration in person identification. Cortex.

[94] Gonzalez I.Q., Leon M.A.B., Belin P., Martinez-Quintana Y., Garcia L.G., Castillo M.S. (2011). Person identification through faces and voices: An ERP study. Brain Res..

