# Supplementary material for: Neural Correlates of Voice Learning with Distinctive and Non-Distinctive Faces
Source: Brain Sci. 2023 Apr 7;13(4):637. doi: 10.3390/brainsci13040637 (PMC10136676; doi:10.3390/brainsci13040637)
Supplement: Supplementary file 1 [file brainsci-13-00637-s001.zip › brainsci-2239428-supplementary.pdf]

Supplemental Tables

Table S1. Summary of statistical analyses (F-values, dfs in parentheses, and effect sizes) of ERP mean amplitudes to faces in the learning phases.

	Faces																	
	P100			N170			P200			N250			LPC			SA (occipito-temporal)		
	F(1,27)	p	η_p^2	F(1,27)	p	η_p^2	F(1,27)	p	η_p^2	F(1,27)	p	η_p^2	F(1,27)	p	η_p^2	F(1,27)	p	η_p^2
Site (S)				83.866	< .001	.756	45.890	< .001	.630	35.020	< .001	.565	27.979	< .001	.509	7.908	.009	.227
Hemisphere (H)	< 1			< 1			< 1			< 1						1.904	.179	.006
Learning Condition (LC)	1.853	.185	.064	14.203	< .001	.345	6.666	.016	.198	12.840	.001	.322	32.545	< .001	.547	14.808	< .001	.354
S x H				< 1			4.396	.046	.140	3.205	.085	.106				< 1		
S x LC				< 1			1.377	.251	.049	1.572	.221	.055	< 1			< 1		
H x LC	< 1			< 1			< 1			< 1						2.617	.117	.088
S x H x LC				1.003	.325	.036	1.764	.195	.061	< 1						< 1		

Table S2. Summary of statistical analyses (F-values, t-values, dfs in parentheses, and effect sizes) of ERP mean amplitudes to voices and faces in the learning phases.

	Voices + Faces										
	N1		P2		SA (occipito-temporal)			SA (fronto-central)			
	t(27)	p	t(27)	p	F(1,27)	p	η_p^2	F	df	p	η_p^2
Site (S)					6.525	.017	.195	20.162	6,162	< .001	.428
Hemisphere (H)					< 1						
Learning Condition (LC)	-0.211	.834	-0.610	.547	4.939	.035	.155	6.685	1,27	.015	.198
S x H					< 1						
S x LC					< 1			1.020	6,162	.322	.036
H x LC					< 1						
S x H x LC					< 1						

Table S3. Summary of statistical analyses (F-values, dfs in parentheses, and effect sizes) of ERP mean amplitudes to voices in the test phases.

	N1			P2			"N250"			SA (occipito-temporal)			SA (fronto-central)			
	F(2,54)	p	η_p^2	F(2,54)	p	η_p^2	F(2,54)	p	η_p^2	F(2,54)	p	η_p^2	F	df	p	η_p^2
Site (S)							10.807	.003	.286	3.796	.062	.123	47.099	6,162	< .001	.636
Hemisphere (H)							6.154	.020	.186	9.800	.004	.266				
Learning Condition (LC)	< 1			1.080	.347	.038	3.357	.042	.111	4.110	.022	.132	2.241	2,54	.116	.077
S x H							3.865	.060	.125	6.208	.019	.187				
S x LC							< 1			< 1			2.051	12,324	.037	.071
H x LC							< 1			< 1						
S x H x LC							< 1			< 1						

Supplemental Figures

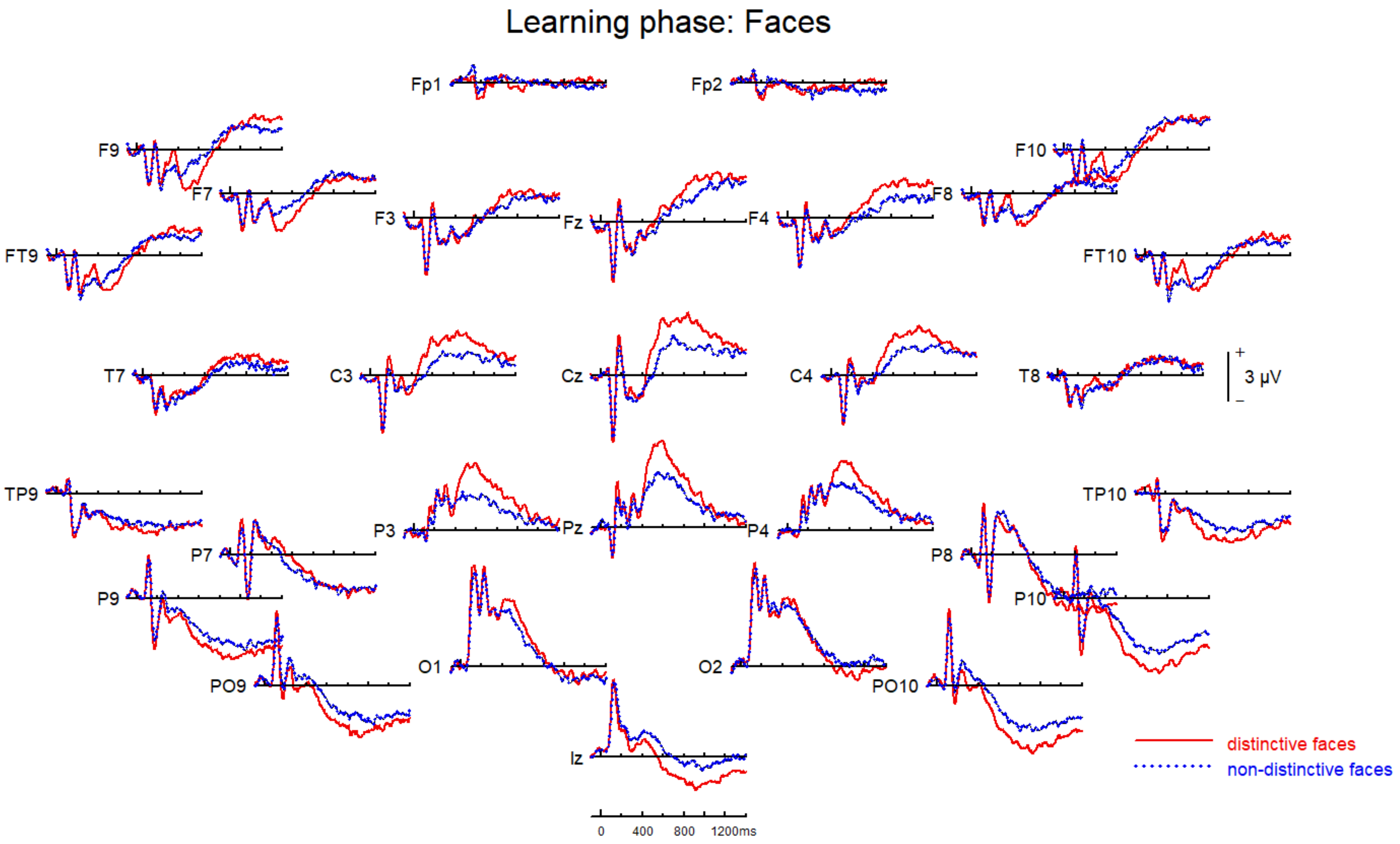

Figure S1. Grand mean averages of face-elicited ERPs in learning phases, shown separately for learning conditions (distinctive or non-distinctive faces) at all 30 electrode sites.

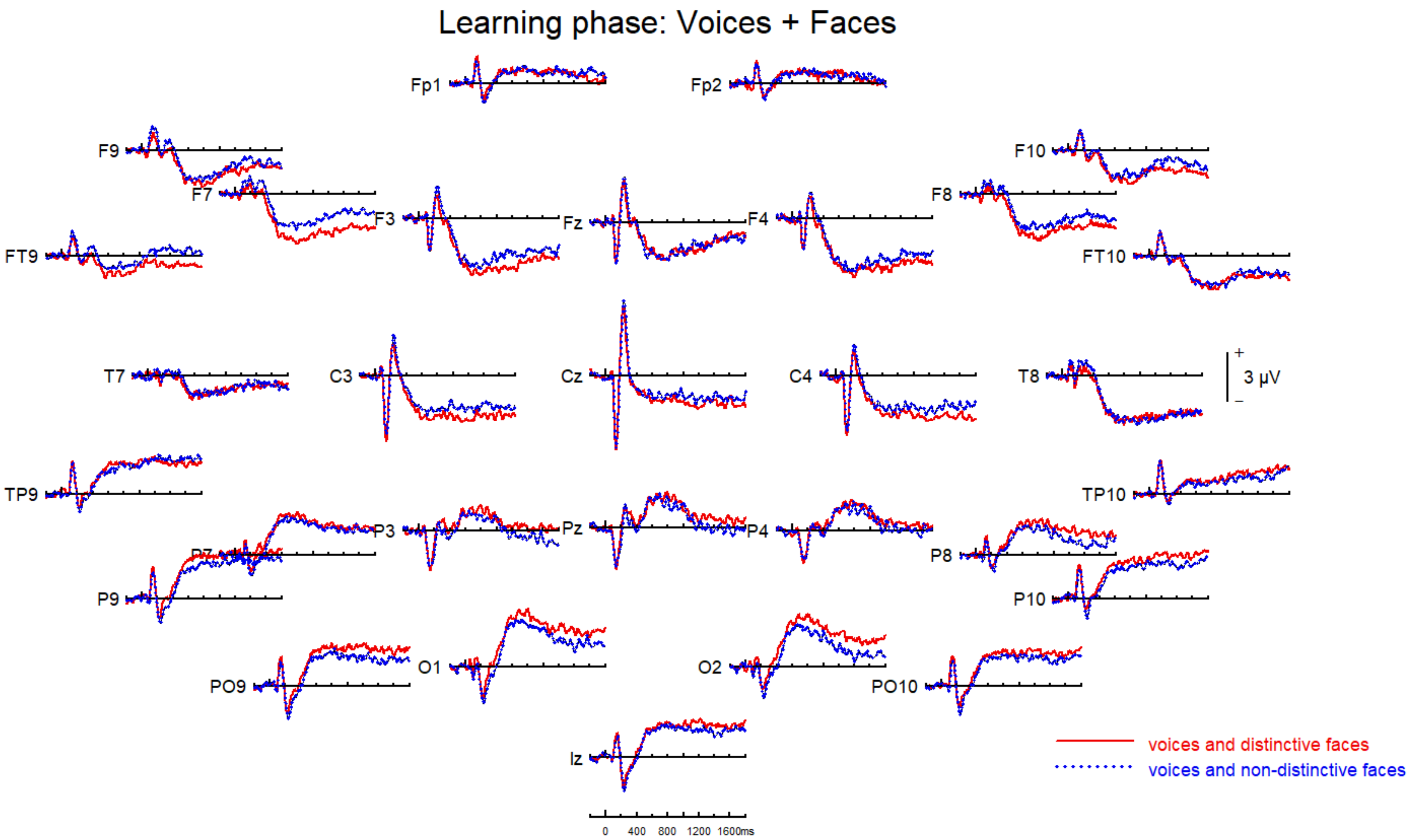

Figure S2. Grand mean averages of voice-elicited ERPs in learning phases, shown separately for learning conditions (concurrent distinctive or non-distinctive faces) at all 30 electrode sites.

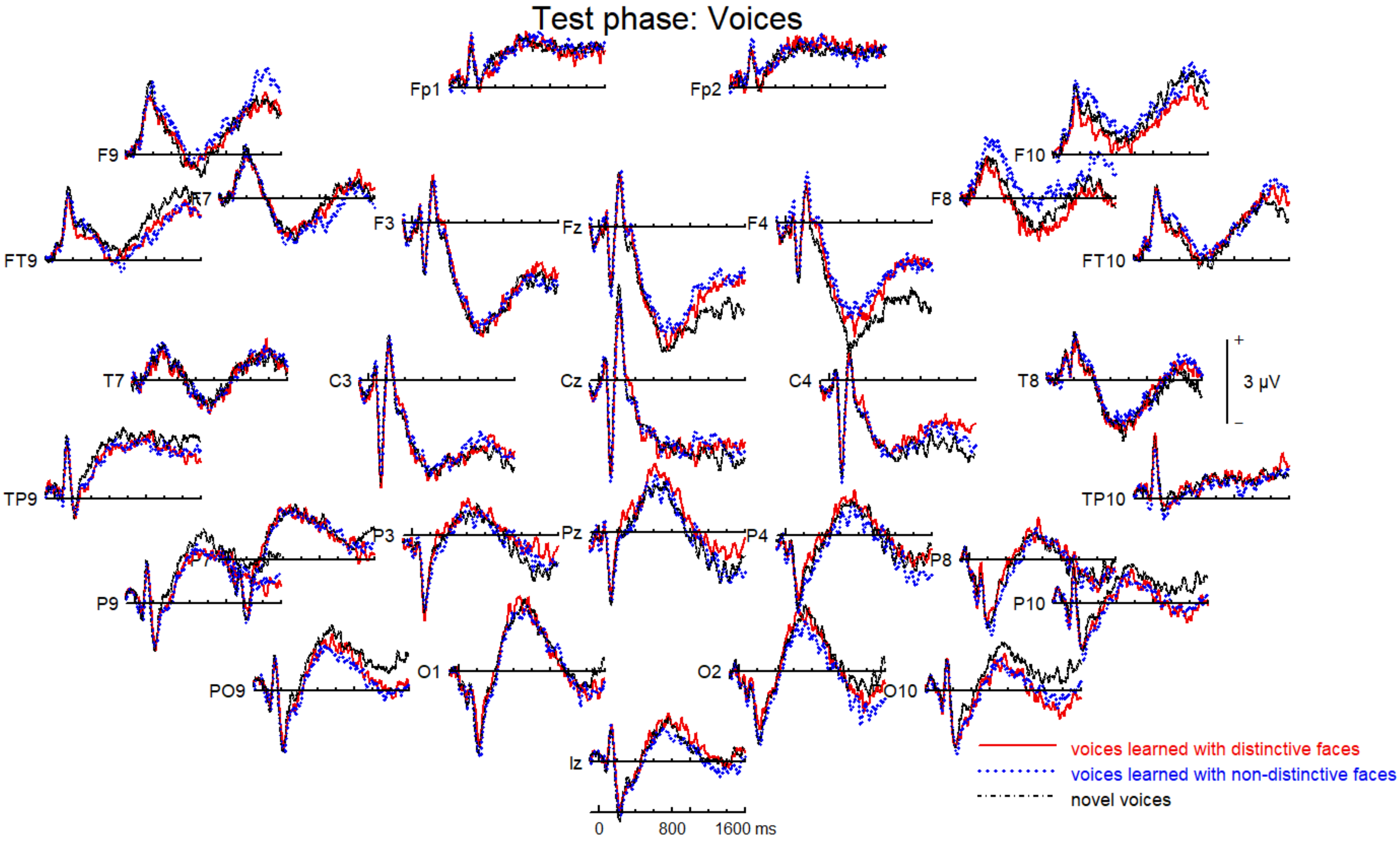

Figure S3. Grand mean averages of voice-elicited ERPs in test phases, shown separately for learning conditions (voices learned with distinctive or non-distinctive faces, novel voices) at all 30 electrode sites.
